# The Popliteofibular Ligament: A Narrative Review of Anatomical Variants and Their Surgical Relevance in Posterolateral Knee Reconstruction

**DOI:** 10.3390/jcm14176322

**Published:** 2025-09-07

**Authors:** Łukasz Olewnik, Ingrid C. Landfald, Bartosz Gonera, Kacper Ruzik, Robert F. LaPrade

**Affiliations:** 1 Department of Clinical Anatomy, Mazovian Academy in Płock, 09-402 Pock, Poland; ingridceciliee@gmail.com (I.C.L.); b.gonera@mazowiecka.edu.pl (B.G.); k.ruzik@mazowiecka.edu.pl (K.R.); 2 VARIANTIS Research Laboratory, Mazovian Academy in Płock, 09-402 Pock, Poland; robertlaprade@tcomn.com; 3 Twin Cities Orthopedics, Edina, MN 55435, USA

**Keywords:** popliteofibular ligament, posterolateral corner, anatomical classification, knee instability, reconstruction, personalized orthopedics, Olewnik

## Abstract

**Purpose:** This review aims to synthesize current knowledge of anatomical variations of the popliteofibular ligament (PFL) and evaluate the clinical relevance of the classification system proposed by Olewnik et al. in the context of the diagnosis, surgical treatment, and rehabilitation of posterolateral corner (PLC) injuries. **Methods:** A comprehensive analysis of anatomical, surgical, and radiological studies concerning the PFL was conducted. The implications of PFL morphological variants were examined across clinical applications, with an emphasis on reconstructive strategies, imaging interpretation, and rehabilitation planning. Emerging research directions, including AI-supported imaging and personalized algorithms, were also explored. **Results:** Olewnik’s classification identifies three distinct types of PFL, each with unique structural and biomechanical properties. Recognizing these variants enhances intraoperative orientation, facilitates tailored surgical techniques, and supports individualized rehabilitation protocols. Variant-specific biomechanics, identified via cadaveric studies and imaging, are essential for optimizing functional outcomes and minimizing postoperative instability. Furthermore, the classification offers a platform for developing future diagnostic and decision-support tools using artificial intelligence. **Conclusions:** The Olewnik et al. classification system should be adopted as a modern anatomical standard for the PFL. Its integration into clinical practice has the potential to improve surgical precision, reduce complication rates, and enhance patient-specific treatment planning. This framework also supports future advancements in orthopedic imaging, education, and AI-driven diagnostics. Beyond descriptive anatomy, we provide a pragmatic surgical algorithm for PLC repair/reconstruction that accounts for scar- and fibrosis-dominated fields and the limited bone stock of the fibular head.

## 1. Introduction

### 1.1. Importance of the Posterolateral Corner (PLC) in Knee Joint Biomechanics

The posterolateral corner (PLC) of the knee is a complex anatomical region that provides essential resistance to varus forces, external tibial rotation, and posterior tibial translation, particularly when the knee is in extension [1,2,3]. Functionally, the PLC serves as a critical static and dynamic stabilizer, acting in concert with the cruciate ligaments and collateral structures to maintain rotational and translational stability during weight-bearing and pivoting activities [4].

Disruption of the PLC, particularly in combination with cruciate ligament injury, can lead to significant biomechanical imbalance and chronic instability, which if left untreated, predisposes the joint to progressive chondral damage, meniscal injury, and early osteoarthritis [5,6]. Restoration of native biomechanics through precise anatomical reconstruction of the PLC has been shown to be essential for regaining full functional capacity and minimizing the risk of graft failure in combined ligament procedures [7,8].

The biomechanical importance of the PLC is further underscored by cadaveric studies that have quantitatively demonstrated the significant role of its individual components, including the fibular collateral ligament (FCL), popliteus tendon, and popliteofibular ligament (PFL), in maintaining joint stability under physiological loading [2,3,9] (Sugita and Amis, 2001; LaPrade et al., 2003; Lasmar et al., 2010). Understanding the interplay among these structures is therefore fundamental in the assessment and management of posterolateral knee injuries.

### 1.2. The PFL as a Key Stabilizing Component

The popliteofibular ligament (PFL) is a distinct anatomical structure within the PLC, originating from the musculotendinous junction of the popliteus tendon and inserting onto the medial aspect of the fibular head. Several anatomical variants have been described, affecting both its origin and insertion [10,11,12,13]. Its biomechanical significance, particularly in controlling external tibial rotation and varus stress, is detailed in Section 2.2.

### 1.3. Diagnostic and Surgical Challenges Arising from Anatomical Variability

The anatomical variability of the PFL complicates both diagnosis and reconstruction. In particular, imaging modalities such as magnetic resonance imaging (MRI) may underrepresent variant forms, contributing to diagnostic ambiguity. A detailed discussion of visualization challenges and modality-specific limitations is provided in Section 3.4.1. From a surgical perspective, classification-based planning is essential to avoid incomplete reconstruction in multi-bundle configurations [6,8,12].

### 1.4. Aim of This Article

Despite growing recognition of the biomechanical relevance of the PFL, its anatomical variability remains insufficiently addressed in diagnostic and surgical protocols. This variation poses challenges for radiologists, orthopedic surgeons, and rehabilitation professionals aiming to restore posterolateral knee function with anatomical precision [14,15].

A recent morphological classification proposed by Olewnik et al. [12] organizes PFL variants into three reproducible types, based on the number and orientation of ligamentous bands. This system offers both anatomical clarity and practical utility supporting improved preoperative imaging, intraoperative identification, and individualized reconstruction strategies.

The objective of this narrative review is to synthesize current knowledge of the anatomical variability of the PFL, with a particular focus on the Olewnik classification. This review explores its embryological background, morphometric features, and clinical relevance in diagnostic, surgical, and rehabilitative contexts. In doing so, it aims to evaluate the classification’s potential as a standardized reference in clinical orthopedics.

However, operative reality rarely mirrors cadaveric footprints: chronic PLC cases are addressed in scarred/fibrotic fields and fixation at the fibular head is constrained by bone stock, making a translational, technique-oriented synthesis essential.

## 2. Anatomy of the Popliteofibular Ligament

### 2.1. Anatomical Location and Relationships with Surrounding Structures

The PFL is a distinct anatomical structure within the PLC of the knee, connecting the musculotendinous junction of the popliteus to the posteromedial surface of the fibular head [2,11,12,13]. It courses obliquely beneath the FCL, forming part of the posterolateral capsular complex. In some anatomical configurations, the PFL merges partially with the arcuate ligament complex, further contributing to posterolateral stabilization [9,10].

The PFL functions as a static stabilizer by linking the popliteus muscle to the fibula, thereby integrating dynamic and ligamentous elements of the PLC. Its anatomical proximity to the FCL and popliteus tendon places it at high risk during injuries involving varus or external rotation forces [2,8]. Awareness of these relationships is essential for accurate identification during surgical exploration and reconstruction.

### 2.2. Biomechanical Function: Control of External Rotation and Varus Stress

The PFL is a critical static stabilizer of the PLC, acting independently to resist external tibial rotation and varus angulation, particularly between 30° and 90° of knee flexion [3,9]. Cadaveric sectioning studies demonstrate that transection of the PFL leads to significant increases in rotational laxity and varus opening, even when the FCL remains intact [6,16], underscoring its non-redundant biomechanical role.

Clinically, failure to reconstruct the PFL adequately, particularly in multiligament knee injuries involving the PCL or FCL, has been associated with persistent instability, suboptimal functional outcomes, and increased revision rates [8,17]. Recognition of this role has led to an increased emphasis on PFL-specific surgical planning, with attention to anatomical variants that may alter biomechanical behavior [12].

### 2.3. Evolution of Anatomical Understanding and Modern Classification Systems

Although historically underrepresented in the anatomical literature and clinical protocols, recent cadaveric and imaging studies have highlighted the PFL as a distinct and morphologically variable structure. These investigations consistently demonstrate variability in the number of ligamentous bundles and their attachment patterns [13,14,18].

Among the classification systems proposed to describe this variability, the three-type model introduced by Olewnik et al. [12] represents a notable advancement. It offers a reproducible framework that facilitates imaging interpretation, surgical planning, and anatomical comparison. The specific anatomical features of this system are discussed in detail in Section 3.1.

## 3. The Olewnik et al. Classification—Structure and Foundations

### 3.1. Anatomical Types

A three-type anatomical classification of the PFL was proposed by Olewnik et al. [12] following the detailed dissection of 137 cadaveric lower limbs. The system distinguishes variants based on the number of ligamentous bands and their distal insertion sites, with the aim of improving clinical identification, imaging interpretation, and surgical planning.

1. Type I—Single Ligament (72.3%)

This was the most prevalent configuration. The PFL originated from the musculotendinous junction of the popliteus muscle and inserted as a single band on the apex of the fibular head. It followed a straightforward oblique course and contributed significantly to PLC stability. Due to its simplicity and subtle imaging appearance, it may be overlooked in minimally invasive procedures or standard MRI protocols [12].

2. Type II—Bifurcated Ligament (8.7%)

This variant shared the same origin as Type I but split distally into two distinct bands:

- The dominant band inserted on the anterior slope of the fibular styloid.
- The smaller band attached to the posterior surface.

The bifurcation creates a more complex biomechanical profile, potentially enhancing rotational restraint but complicating imaging assessment and surgical reconstruction.

3. Type III—Double Ligament (7.3%)

The rarest form consisted of two entirely separate ligaments:

- One originated from the popliteus tendon and inserted anteriorly.
- The other arose from the musculotendinous junction and inserted posteriorly on the fibular styloid.

This dual-band configuration may provide increased rotational stability but presents higher intraoperative complexity. If one of the bands is not identified and reconstructed, functional outcomes may be compromised.

This classification provides a clinically meaningful framework for understanding PFL variability and supports its integration into preoperative planning and intraoperative decision-making [12].

### 3.2. Morphometry and Attachment Points

#### Differences in the Course, Length, and Width of Individual Bundles

The morphometric characteristics of the PFL exhibit notable variability among its anatomical types. In a cadaveric study of 137 lower limbs, Olewnik et al. [12] provided detailed measurements for each variant.

Type I is characterized by a short, single oblique band extending from the musculotendinous junction of the popliteus to the fibular head apex. It measured 8.72 ± 1.91 mm in length, with proximal and distal widths of 2.99 ± 1.36 mm and 3.10 ± 1.48 mm, respectively.

Type II displays a bifurcated distal course. The dominant lateral band measured 4.17 ± 0.69 mm, and the medial band measured 7.36 ± 1.40 mm. The different structure may modulate rotational resistance differently from Type I.

Type III comprises two entirely separate ligamentous bands. The main band averaged 8.72 mm in length and the accessory band was 7.95 mm. Proximal widths were 2.99 mm and 1.92 mm, and distal widths were 3.10 mm and 1.56 mm, respectively.

Sex-based comparisons revealed significantly larger dimensions in males, particularly in distal width and thickness (*p* < 0.01), with no significant side-to-side asymmetry.

These morphometric distinctions underscore the relevance of the Olewnik classification in preoperative planning and anatomical reconstruction.

### 3.3. Frequency and Embryological Relevance

The prevalence and embryological origins of PFL variants have important diagnostic and clinical implications. As detailed in the morphometric analysis (see Section 3.2), Olewnik et al. [12] identified the PFL in 88.3% of 137 dissected knees, with Type I being most common. Embryologically, the variants likely reflect incomplete regression or divergence of fetal myotendinous precursors analogous to the variability observed in the palmaris longus or plantaris tendon [19,20,21]. These findings support the classification’s dual role as a descriptive and developmental tool (Table 1).

Among all existing descriptions, Olewnik et al. [12] provide the most detailed and clinically applicable classification of the PFL. Unlike earlier works, which describe its existence or general morphology, this system identifies distinct anatomical variants based on origin, course, and insertion patterns. These distinctions are particularly valuable for preoperative planning and intraoperative strategy, especially in complex posterolateral corner (PLC) reconstructions. The inclusion of Types II and III, which are often overlooked, directly informs surgical decisions regarding graft number, trajectory, and fixation. In comparison, previous studies either oversimplify the anatomy (e.g., single vs. double band) or lack the operative context altogether.

### 3.4. The Classification as a Clinical Tool

#### 3.4.1. Potential for Standardized MRI Descriptions

MRI remains the primary modality for evaluating the soft tissue components of the PLC; however, consistent identification of the PFL remains challenging due to its small caliber, deep anatomical position, and morphological variability. The classification introduced by Olewnik et al. [12] provides a structured reference that may improve radiologic assessments by standardizing the description of PFL subtypes and insertion patterns.

Recent work by Heylen et al. [22] demonstrated poor interobserver agreement in identifying the PFL on MRI, particularly for less common configurations. Incorporating a consistent typology may enhance diagnostic reliability across institutions, especially when evaluating complex variants that deviate from linear or single-band morphologies.

#### 3.4.2. Recommendation for Integration into Orthopedic Practice

Preoperative recognition of PFL anatomy is a critical component of achieving anatomical fidelity in PLC reconstruction. The classification proposed by Olewnik et al. [12] provides a practical framework that guides individualized surgical strategies, including graft choice, tunnel trajectory, and fixation configuration.

Failure to adapt reconstruction techniques to the specific PFL variant may contribute to suboptimal outcomes, including unresolved instability [8]. Incorporating the classification into preoperative protocols such as MRI reporting, surgical planning documents, and intraoperative checklists can enhance procedural accuracy and reduce the likelihood of revision, particularly in complex or multiligamentous cases.

#### 3.4.3. Educational and Surgical Planning Utility

In addition to its clinical relevance, the Olewnik classification holds significant value in anatomical education and surgical simulation. It offers a reproducible system for teaching PFL morphology across dissection courses, radiologic interpretation workshops, and orthopedic curricula. Its typological clarity also facilitates interdisciplinary communication among anatomists, radiologists, and surgeons.

Furthermore, this classification can serve as a foundation for surgical templating, biomechanical modeling, and algorithmic planning in reconstruction procedures, aligning with the principles of personalized and precision orthopedics [12].

## 4. Clinical Significance of PFL Anatomical Variants

### 4.1. Correlation Between the PFL Type and Clinical Symptoms: Rotational Instability, Posterolateral Pain, and Sense of the Knee “Giving Way”

Anatomical variability of the PFL has a direct impact on the clinical presentation of posterolateral knee dysfunction. Different PFL types may be associated with distinct patterns of rotational instability, pain, and subjective symptoms, particularly in complex PLC injuries.

In Type I (single-band), the limited redundancy of stabilizing fibers may predispose the knee to subtle but persistent rotational instability. Patients frequently describe a sensation of the knee “giving way”, particularly during pivoting activities, descending stairs, or walking downhill [3,6]. These symptoms correlate with the PFL’s mechanical role in resisting external rotation and varus force, as discussed in Section 2.2.

Type II (bifurcated) features two distal insertions that may enhance load distribution. However, the smaller posterior band is often underappreciated on imaging and may be missed intraoperatively. Isolated injury to this band can manifest as focal posterolateral pain, while clinical signs of instability become apparent primarily when both bands are affected [12].

Type III (double PFL) comprises two anatomically distinct bundles, potentially offering greater resistance to external tibial rotation. Partial damage involving only one of these structures may be clinically occult unless high-resolution imaging or arthroscopy is employed. In contrast, complete disruption, especially when combined with injury to the FCL or popliteus tendon, can result in significant rotational laxity and instability [7,8].

Recognition of these type-specific symptom patterns supports the accurate clinical interpretation of diagnostic maneuvers such as the dial test, external rotation recurvatum test, and posterolateral drawer test. Without anatomical awareness, subtle instability associated with rare PFL variants may go undiagnosed, particularly in low-grade or partial injury scenarios.

### 4.2. Association of Reconstruction Failure with Unrecognized or Incomplete Restoration of PFL Bundles

Failure to recognize or adequately restore the anatomical complexity of the PFL has emerged as a significant factor in the failure of PLC reconstruction. In many cases, the persistence of rotational instability, graft elongation, or mechanical dysfunction is attributable not to technical error per se, but rather to the incomplete reconstruction of all functionally relevant PFL components [12].

Traditional reconstruction techniques frequently assume a single-band configuration of the PFL, despite increasing evidence that multiple anatomical variants, such as bifurcated or dual-bundle forms, are not only prevalent but biomechanically consequential (see Section 3.1). Neglecting these structural differences can lead to an asymmetric load distribution and persistent instability under varus or rotational stress [8].

Biomechanical data further support this concern. Isolated reconstruction of the FCL, without concomitant PFL restoration, fails to fully re-establish posterolateral stability [8]. Similarly, instability may persist following technically accurate FCL and cruciate ligament reconstruction if complex PFL variants are unrecognized or inadequately reconstructed [7].

To mitigate these risks, the integration of standardized classification systems, such as the one proposed by Olewnik et al. [12], into preoperative protocols and intraoperative checklists is recommended. Such integration enables tailored graft strategies, optimized tunnel placement, and type-specific fixation angles, improving surgical precision and outcomes, particularly in chronic or multiligament-injured knees.

### 4.3. Possible Coexistence of PFL Types with Other Posterolateral Anatomical Variations

Anatomical variability within the PLC is not limited to the PFL alone. As emphasized by Olewnik et al. [12], specific PFL types may coexist with morphological variants of the FCL, popliteus tendon, or arcuate complex, complicating both diagnosis and surgical planning.

For instance, Type III PFL configurations may overlap spatially with bifid or accessory popliteus tendons, increasing the risk of intraoperative misidentification or unintentional injury [12]. Likewise, variations in the course or insertion of the FCL may obscure smaller or atypical PFL bands during imaging or surgery [2,10].

Additional complexity arises from nearby structures such as the arcuate or fabellofibular ligaments, which may share anatomical territories with variant PFL configurations. These overlapping elements suggest that PLC stability relies on an integrated system of interdependent components.

Therefore, diagnostic and surgical evaluations of the PFL should not occur in isolation. A comprehensive, classification-based assessment of the entire PLC is essential to ensure complete anatomical reconstruction and avoid overlooked contributors to instability.

### 4.4. Clinical Examination Relevance—Importance of Stress Testing and the Dial Test in Suspecting Specific PFL Types

While imaging and intraoperative findings are crucial in evaluating PLC injuries, clinical examination remains an indispensable first-line tool in screening for PFL-related pathology, particularly when anatomical variants are suspected. The interpretation of stress-based tests, especially the dial test, gains additional diagnostic value when analyzed through the lens of PFL typology.

The dial test, performed with the patient in the prone or supine position at 30° and 90° of knee flexion, is designed to detect increased external tibial rotation indicative of PLC insufficiency. In the context of Type I PFL, increased asymmetry at 30° flexion, especially without posterior drawer positivity, may reflect isolated PFL compromise [6,9]. Due to the relatively simple and singular structure of Type I PFL, even partial tearing may result in clinically detectable rotational laxity.

In contrast, Type II and III PFL variants, characterized by a bifurcated or dual-ligament anatomy, may not demonstrate overt laxity unless multiple components are injured. The presence of subtle but consistent increases in external rotation, combined with tenderness over the fibular head or posterolateral capsule, should raise suspicion of a complex PFL morphology or partial disruption of deeper fibers not easily captured on standard imaging [12]. These types may also yield false-negative stress tests if one bundle remains intact, masking functional instability.

Other clinical maneuvers, such as the external rotation recurvatum test, posterolateral drawer test, and reverse pivot shift, may help distinguish isolated PFL pathology from more extensive PLC injuries. Still, these tests lack specificity and should be interpreted in the context of detailed anatomical knowledge, ideally augmented by classification-based reasoning.

As emphasized by Chahla et al. [7], a clinical examination is most powerful when integrated with patient history, mechanism of injury, and imaging findings. Recognizing that different PFL types may produce variable clinical signatures, the examiner should be prepared to adjust interpretive thresholds based on the suspected underlying morphology.

In this context, the Olewnik classification can serve not only as a surgical and radiological guide but also as a conceptual framework during physical assessments, especially in complex or revision cases where imaging may be inconclusive.

### 4.5. Anatomical Awareness Aids in Differential Diagnosis Between PFL and Injuries of the FCL, Popliteus, or Arcuate Complex

An accurate differential diagnosis of posterolateral knee instability requires an understanding of the anatomical interplay between the PFL, FCL, popliteus tendon, and arcuate complex. Because of their spatial proximity and biomechanical overlap, injury patterns can be misinterpreted without adequate anatomical awareness. Recognizing specific PFL variants, especially Types II and III, may help differentiate isolated PFL pathology from FCL or popliteus dysfunction based on the symptom distribution and functional tests [7,8,9]. The classification by Olewnik et al. [12] provides a framework to support this process. A comprehensive discussion of imaging strategies is presented in Section 3.4.1.

## 5. Surgical and Orthopedic Relevance

### 5.1. Preoperative Significance

#### 5.1.1. Identification of the PFL Type as a Component of PLC Reconstruction Planning

Accurate identification of the PFL type is a crucial step in preoperative planning for PLC reconstruction, especially in revision or multiligament procedures. The classification by Olewnik et al. [12] informs graft configuration and tunnel placement tailored to the patient’s native anatomy.

High-resolution imaging, ideally interpreted with reference to classification-based criteria, enhances the detection of complex PFL variants. For a detailed analysis of imaging limitations and optimization strategies, see Section 3.4.1. Integrating PFL typing into surgical planning supports precise, anatomy-guided reconstruction.

#### 5.1.2. Translational Constraints: Scar/Fibrosis and Fibular Head Bone Stock

Real-world PLC surgery is frequently undertaken in scarred/fibrotic fields, where native insertions of the PFL are obscured and footprint-literal repair can be unreliable; a pragmatic, vector-based plan is therefore advisable [7,23,24]. Limited and variably shaped bone stock at the fibular head further constrains the tunnel choice and spacing, increasing the risk of blowout if overcrowded [2,9]. Preoperative mapping should anticipate variant anatomy and the peroneal nerve course, with a clear fallback to onlay/anchor or single-tunnel constructs if the tissue or bone quality is suboptimal [15,24].

#### 5.1.3. Age- and Sex-Related Considerations (Qualitative)

Age-related changes in enthesis and bone mineral density, and sex-related dimorphism in morphometry plausibly influence graft sizing, tunnel diameter/spacing, and the fixation method, warranting patient-specific tailoring where feasible [2,15]. Imaging can aid in this tailoring MRI for soft-tissue quality and CT when a bony geometry is pivotal, while recent work underscores both the feasibility and challenges of identifying the PFL on imaging [14,22,25]. Our dataset does not support powered age- or sex-stratified inference; these implications remain qualitative and require prospective validation [7].

### 5.2. Impact of Variants on Surgical Technique Selection

The anatomical classification by Olewnik et al. [12] provides a practical foundation for tailoring surgical techniques to the specific morphology of the PFL. Each variant may require a distinct reconstructive approach to fully restore posterolateral knee stability.

Although Type I configurations offer relatively straightforward reconstruction, their deep location and fine caliber make them susceptible to intraoperative oversight, especially in arthroscopic procedures. More complex variants, such as bifurcated or dual-ligament types, may involve multiple insertions or fascicles that require targeted grafting strategies. These patterns are discussed in detail in Section 3.1 and have been shown to influence rotational control and postoperative outcomes [15,25].

Standard single-bundle techniques may be insufficient in patients with Type II or III variants. In such cases, dual tunnel placement or multi-anchor constructs may be necessary to replicate native biomechanics [8,17]. Failure to identify and restore these complex morphologies can lead to undercorrection, increased graft strain, and an elevated revision risk.

Adopting a classification-based approach throughout the surgical workflow from MRI analysis to intraoperative dissection facilitates a more precise and individualized reconstruction, ultimately improving long-term outcomes and minimizing technical failure.

#### Scar Tissue and Fibrosis: Why Footprints ≠ Fixation Sites

Chronic PLC injuries often generate broad adhesion bands that create non-anatomic “stability zones”; so single-point fixation to a theoretical footprint may fail to restore the functional vector [23,24]. In such fields, selective preservation of the scar that supports the desired line-of-force, release of the scar that tethers maldirection, and multi-point soft-tissue capture or onlay techniques are preferable when native entheses are indistinct [6,26]. This vector-driven approach aligns with anatomic principles while reflecting operative reality [7].

### 5.3. Modifications of Reconstruction Techniques

#### 5.3.1. Adjustment of Tunnel Placement and Graft Selection

Recognition of anatomical variability within the PFL necessitates the adaptation of standard PLC reconstruction techniques, particularly in terms of tunnel trajectory, fixation points, and graft selection. While traditional reconstructions often use a single fibular tunnel directed toward the anterior slope or apex of the fibular head, this may be inadequate for more complex PFL variants involving multiple or separate insertions [8,12].

In bifurcated configurations, tunnel placement targeting only the anterior insertion may neglect posterior fascicles. Dual-angle tunnel systems or separate anchors may therefore be required to replicate the anatomical tension distribution [17,26]. In dual-band morphologies, reconstructive strategies may involve two independent tunnels or cortical suspensory fixations to restore physiological vector alignment.

Graft selection should be tailored accordingly. Thinner autografts such as gracilis tendon may suffice in single-band cases, while thicker or multiple grafts, e.g., semitendinosus, quadriceps, or dual constructs, may be necessary in multi-band configurations. The graft diameter, orientation, and tensioning must align with the native anatomical course to avoid overconstraint or ineffective stabilization [6,9].

#### 5.3.2. Opportunity to Develop Reconstruction Strategies Dedicated to Specific PFL Types

The Olewnik classification [12] provides a foundation for designing reconstruction strategies tailored to specific anatomical variants of the PFL. As in cruciate ligament surgery, individualized approaches may yield superior outcomes in PLC procedures. For instance,

- Type I configurations may be addressed with single-tunnel reconstructions and straightforward grafts targeting the fibular apex.
- Type II variants may benefit from Y-shaped grafts or dual-suture anchors to restore both bifurcated insertions.
- Type III cases may require two independent grafts with separate fixation and individualized tensioning, replicating the dual-band architecture [12,18,24].

Future surgical protocols should be informed by morphometric validation and biomechanical modeling based on classification-aware designs. The integration of 3D navigation, intraoperative imaging, and patient-specific instrumentation (PSI) could further enhance graft placement accuracy and functional outcomes.

By applying classification-guided techniques, surgeons may improve posterolateral stability, reduce revision rates, and restore more natural joint kinematics.

#### 5.3.3. Fibular Head Constraints (Limited Bone Stock) and Fixation Strategy

The fibular head’s limited bone stock and variable geometry constrain multi-tunnel strategies and demand generous spacing to preserve cortical bridges [2,9]. Where bone is borderline, a single oblique tunnel with suspensory fixation, a suture–anchor onlay, or a fibular-based sling can restore varus/external rotation restraint while minimizing the fracture risk [23,26]. The technique should also reflect the PFL’s contribution to PLC stability reconstruction that reproduces the PFL vector and improves rotational control, including when a tibial tunnel is required for anatomic restoration [8,17]. Throughout the process, meticulous identification and protection of the peroneal nerve are mandatory [15,24].

### 5.4. Arthroscopy and Intraoperative Identification

#### Visualization and Differentiation Difficulties Depending on the Type

Arthroscopic identification of the PFL remains technically challenging due to its deep anatomical location, small caliber, and morphological variability. These factors are particularly relevant in minimally invasive PLC reconstructions, where precise localization is critical for accurate graft placement.

Single-band configurations may be difficult to detect arthroscopically, especially in the presence of synovitis or hemorrhage. In such cases, the PFL may be misinterpreted as capsular thickening or a fascial structure [9,17]. More complex variants, including bifurcated or dual-band types, often require targeted exploration to visualize posterior or accessory insertions regions not easily accessible with standard instrumentation [12,25].

Misidentification or partial exposure of variant PFL bands can lead to incomplete reconstruction or non-anatomical tunnel placement, even when the surgical technique is otherwise sound [2,8]. In Type III variants in particular, accessory bands may be mistaken for components of the arcuate ligament or popliteus tendon.

To mitigate these challenges, advanced techniques, such as arthroscopic-assisted open dissection, enhanced posterolateral portals, and intraoperative navigation or fluoroscopy, should be considered. In revision or complex cases, intraoperative ultrasonography and preoperative 3D reconstruction may further assist in identifying poorly visualized structures.

Incorporating classification-based expectations into surgical planning can enhance the intraoperative orientation, allowing the surgeon to anticipate the likely anatomical configuration and avoid overlooking critical stabilizing components.

### 5.5. Clinical Consequences of Technique Mismatch to the PFL Variant

#### 5.5.1. Persistent Rotational Instability Despite Anatomical PLC Reconstruction

Persistent rotational instability following anatomically intended PLC reconstruction is a common and frustrating complication. In many cases, this results not from technical error but from a failure to recognize the full anatomical complexity of the PFL, particularly in multi-bundle configurations [12].

The omission of secondary PFL components often present in Types II and III may lead to residual laxity, despite an apparently complete reconstruction [7,8]. This is particularly problematic in high-demand patients, where even subtle deficits may impair function or graft longevity [6].

The underlying biomechanical mechanism is described in Section 2.2, where the role of the PFL in controlling external tibial rotation and varus stress is emphasized. Preoperative planning informed by high-resolution imaging and classification-based assessment remains essential to ensure the restoration of all biomechanically relevant structures [9,12].

#### 5.5.2. Misidentification May Overload Adjacent Structures

Inadequate reconstruction of variant PFL anatomy may result in compensatory overloading of adjacent stabilizers, particularly the FCL and popliteus tendon. These structures, although critical in PLC mechanics, are not designed to fully compensate for unaddressed PFL function [2,26].

Such overload may manifest as posterolateral pain, early graft elongation, or secondary failure, especially if rotational loads are misdirected onto the popliteus tendon [9,24]. In the setting of multiligament reconstruction, the resulting imbalance may also jeopardize the ACL or PCL grafts through non-physiological motion [8].

#### 5.5.3. Increased Risk of Revision Surgery

Unrecognized PFL variants are a significant but underappreciated factor in the failure of PLC reconstructions. Even when grafts are correctly placed, omitting one of the stabilizing bands may lead to the recurrence of instability symptoms and patient dissatisfaction [6].

Revision rates remain higher in PLC procedures compared to isolated cruciate reconstructions, in part due to the difficulty of restoring rotational control [8]. Patient-perceived failure may occur despite radiographic or manual stability, especially if sport-specific limitations persist.

To reduce the revision risk, individualized surgical strategies informed by a detailed anatomical classification should be routinely implemented [12].

#### 5.5.4. Mismanagement of Type III Variants

Type III PFL variants, characterized by two distinct ligaments, present unique technical challenges. Attempting to address these complex structures with single-bundle reconstruction techniques can lead to misplacement of tunnels, suboptimal tensioning, and incomplete restoration of function [2,12].

Incorrect graft positioning in Type III knees may alter joint isometry and create an uneven force distribution, contributing to early graft fatigue and failure, despite otherwise technically correct procedures [9,26].

Preoperative recognition of this configuration is critical. When identified, surgeons should consider dual-bundle techniques or modified tunnel systems to ensure biomechanical fidelity.

#### 5.5.5. Importance of Documenting the PFL Type as a Quality Indicator

Explicit intraoperative documentation of the PFL type based on the Olewnik classification should be considered a marker of procedural quality in modern PLC surgery [12]. It reflects anatomical awareness, enables continuity of care, and improves reproducibility in revision scenarios [8].

Such documentation also contributes to larger datasets linking variant anatomy to outcomes, supporting future research and evidence-based refinements in surgical technique. As classification-based surgery evolves, this form of structured reporting may also influence clinical benchmarking and reimbursement criteria.

In this context, the PFL classification should be incorporated into operative checklists and postoperative summaries as part of standard best practice in posterolateral knee reconstruction.

#### 5.5.6. Clinical Algorithm and Technical Pearls

**Figure 1:**
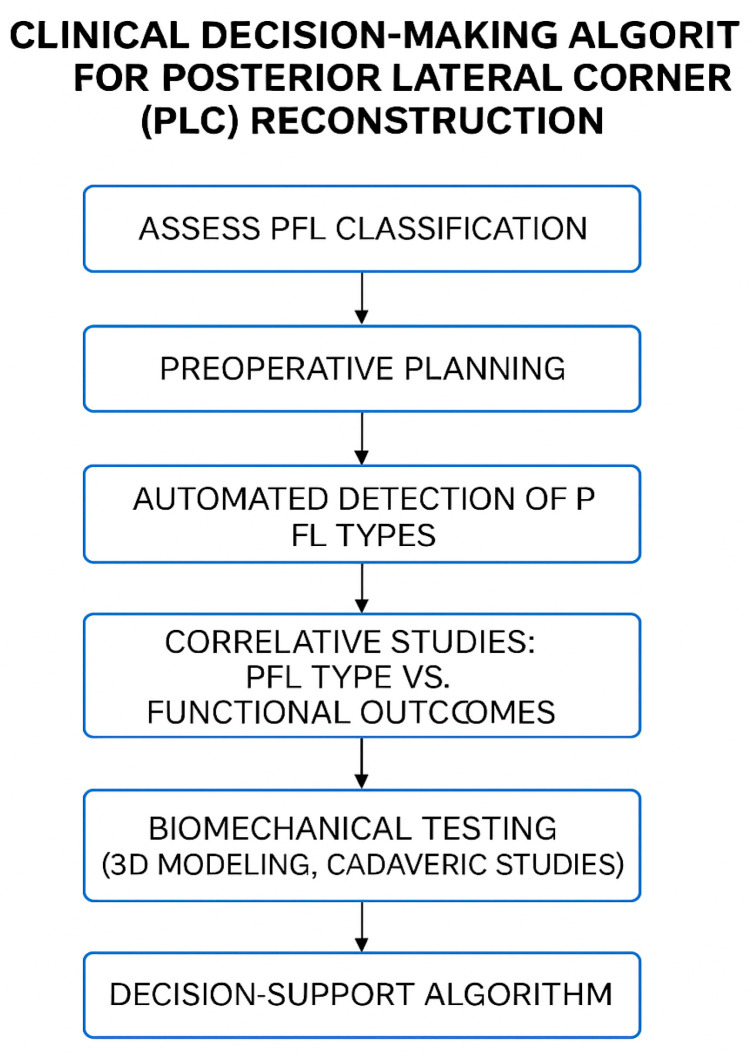
Clinical decision-making algorithm for posterolateral corner (PLC) reconstruction based on the popliteofibular ligament (PFL) classification. The flowchart outlines the integration of anatomical variant identification into surgical planning, incorporating steps such as classification assessment, preoperative planning, AI-supported detection, outcome correlation, and biomechanical validation.

Practical pearls for PLC reconstruction incorporating the identified PFL type (see Figure 1) are as follows:

- Identify and protect the common peroneal nerve early; re-check after drilling [15,24].
- Debride to recreate planes, but avoid removing the scar that supports the desired vector [7,23].
- With limited fibular bone stock, favor a single oblique tunnel, anchor onlay, or sling construct; avoid tunnel overcrowding [2,9].
- Reproduce vectors and physiologic length change rather than chase indistinct footprints; consider graft augmentation in poor-quality tissue [6,26].
- Maintain generous tunnel spacing on the fibular head to preserve cortical bridges [2,9].
- Confirm stability dynamically through range of motion (ROM) before final fixation; adjust tension accordingly [6,23].
- Use intraoperative fluoroscopy when the tunnel trajectory or purchase is uncertain [24].
- Document the PFL type intraoperatively to aid in rehabilitation planning and future audits; imaging reliability data support systematic recognition and reporting [12,14,27].

## 6. Relevance in Rehabilitation and Conservative Management

### 6.1. Tailoring Rehabilitation Programs Based on the PFL Variant and Stabilization Method

The PFL, as categorized by Olewnik’s anatomical classification, displays marked morphological diversity that has direct implications for individualized rehabilitation planning. Rehabilitation protocols following PLC injuries or surgical reconstruction must consider the specific PFL variant identified preoperatively or intraoperatively [12]. Variants with robust or duplicated structures (e.g., Types II and III) may offer greater passive restraint to external rotation, permitting more confidence in early controlled mobilization. In contrast, small-caliber or hypoplastic presentations—most often within the Type I spectrum—may warrant a more cautious progression in dynamic and load-bearing exercises to avoid exacerbating instability [6,12,23]. Moreover, the type of stabilization method, be it anatomical graft reconstruction, ligament augmentation, or conservative bracing, modifies loading across the PLC and should guide the rehabilitation algorithm [16].

### 6.2. The Role of Anatomical Knowledge in Assessing the Risk of Recurrent Instability

Precise anatomical characterization of PFL variants improves risk stratification for recurrent instability in both conservatively and surgically managed patients. Atypical configurations or the absence of fibular attachment can result in persistent posterolateral laxity if not addressed appropriately, especially in high-demand populations [11,12,16]. Imaging modalities such as MRI or high-resolution ultrasound play key roles in delineating these variants and informing treatment planning. For example, a bifid PFL may produce misleading signs of stability during clinical testing while remaining biomechanically insufficient under load [11,12]. Such insights help clinicians anticipate treatment failure or reinjury and adjust care plans proactively.

### 6.3. Monitoring the Response to Conservative Treatment in the Context of the Individual PFL Anatomy

Successful conservative management of isolated or low-grade PLC injuries is contingent on an appreciation of PFL anatomical variability. Individuals with well-developed and biomechanically effective PFL variants tend to respond better to structured rehabilitation and functional bracing [16]. Regular clinical assessment using dial tests and external rotation recurvatum signs, coupled with functional scoring and periodic imaging, allows for accurate monitoring of progress. When improvement is limited despite adherence to rehabilitation protocols, particularly in patients with anatomically compromised variants, surgical evaluation should be reconsidered. Integrating anatomical insights into follow-up evaluations is therefore essential for guiding conservative treatment decisions.

In view of these translational considerations, we outline targeted avenues for research that can validate PFL-type-based planning and refine fixation choices in real-world, scar-dominant PLC surgery.

## 7. Future Research Directions

### 7.1. Correlative Studies: PFL Type vs. Functional Outcomes After PLC Reconstruction

Future research should prioritize well-designed clinical studies that examine the correlation between specific types of the PFL, as defined by Olewnik’s classification, and postoperative outcomes following PLC reconstruction. Determining whether certain PFL variants are associated with improved stability, lower reoperation rates, or quicker functional recovery would enable the development of more personalized treatment protocols [12]. To date, such correlations have not been comprehensively explored, marking a significant gap in the orthopedic literature.

### 7.2. Biomechanical Testing of PFL Types—3D Modeling and Cadaveric Studies

Advancing our biomechanical understanding of PFL variations is critical. This may be achieved through integrated approaches combining three-dimensional (3D) finite element modeling with cadaveric dissection and loading simulations. Such methods can quantify how each PFL variant contributes to the rotational and varus stability of the knee and assess the mechanical implications of reconstruction techniques tailored to specific PFL morphotypes [23].

### 7.3. Potential for the Automated Detection of PFL Types in Imaging Using Artificial Intelligence

The rapid evolution of artificial intelligence (AI) in musculoskeletal imaging provides new opportunities for the automated classification of PFL variants on MRI or high-resolution ultrasound. Machine learning algorithms trained on large, annotated datasets could assist radiologists and surgeons in the preoperative identification of PFL types, especially in complex or subtle presentations, enhancing diagnostic precision and reducing inter-observer variability.

### 7.4. Development of Decision-Support Algorithms Incorporating PFL Classification in Preoperative Planning

The integration of anatomical classification systems such as Olewnik’s into digital decision-support tools holds promise for surgical planning (see Figure 1). By incorporating imaging-based recognition of PFL variants and correlating them with patient-specific risk profiles, such systems could offer evidence-based recommendations for reconstruction techniques and postoperative rehabilitation, ultimately improving clinical outcomes [11,12].

## 8. Limitations

While this review integrates anatomical, radiological, biomechanical, and surgical perspectives on the PFL, several limitations must be acknowledged. First, the core classification by Olewnik et al. [12] is grounded in cadaveric dissection and has not yet been validated in large prospective clinical cohorts. Second, evidence on variant-specific reconstruction outcomes remains limited, particularly for less common Type II and III configurations. Third, imaging-based recognition of PFL types—though improving—still lacks standardized radiological criteria across institutions. In addition, our dataset did not support age- or sex-stratified inferences; demographic variables were either unavailable or underpowered. Importantly, real-world PLC surgery is performed in scarred/fibrotic fields and is constrained by the fibular head’s limited bone stock—conditions that reduce the direct translatability of idealized cadaveric/MRI footprint coordinates. These realities motivated the operative, vector-based algorithm provided here. Finally, we did not quantitatively assess fibular head bone quality or tunnel failure risk, which warrants targeted imaging and biomechanical evaluation.

## 9. Conclusions

The anatomical classification of the popliteofibular ligament (PFL) proposed by Olewnik et al. represents a significant advancement in the understanding of posterolateral knee anatomy. Given its detailed morphological categorization and practical relevance, this system should be regarded as a contemporary standard for describing PFL anatomy.

Its systematic implementation in clinical settings may enhance diagnostic precision, improve surgical planning, and reduce the incidence of reconstruction-related complications. Furthermore, its clarity and reproducibility offer valuable benefits in medical education and training.

Finally, this classification provides a foundational framework for the development of a modern, personalized approach to posterolateral knee management—one that integrates anatomical variation with individualized treatment strategies and emerging technologies such as AI-based imaging and biomechanical modeling.

Translating PLC anatomy to the operating room requires acknowledging two pervasive constraints: scars/fibrosis that obscure native insertions and the limited bone stock of the fibular head. A vector-based approach, supported by single-tunnel/anchor or sling options when bone is borderline, may achieve more reliable varus–external rotation control than footprint-literal repair in chronic cases. Our clinical algorithm and table are intended to standardize these decisions.

## Figures and Tables

**Table 1:** Comparative overview of anatomical classifications of the popliteofibular ligament (PFL).

Author/Source	Number of Types	Classification Criteria	Distal Insertion	Structural Variability Included	Clinical Relevance
Olewnik et al. [12]	3	Number of bands, origin, and insertion points	Type I—apex of the fibular head; Type II—bifurcation with dual insertions; Type III—two separate ligaments	Yes	High—guides graft selection, tunnel placement, and reconstruction planning
Maynard et al. [18]	None	General morphology and localization	Fibular head (not further differentiated)	Limited	Moderate—identifies the PFL as a key stabilizer but lacks practical application
Wadia et al. [13]	2	Number of bands (single vs. double)	Apex vs. bifurcated insertion	Yes—basic level	Moderate—highlights imaging and surgical implications
Covey [1]	None	Presence vs. absence	Fibular head	No	Moderate—emphasizes a role in external rotation control

## List of Abbreviations

ACL	Anterior Cruciate Ligament
ALL	Anterolateral Ligament
CT	Computed Tomography
DL	Deep Learning
FCL	Fibular Collateral Ligament
LCL	Lateral Collateral Ligament
LM	Lateral Meniscus
MCL	Medial Collateral Ligament
MRI	Magnetic Resonance Imaging
PA	Popliteal Artery
PFL	Popliteofibular Ligament
PLC	Posterolateral Corner
PLT	Popliteus Tendon
PM	Popliteus Muscle
PTFL	Posterior Talofibular Ligament
US	Ultrasound
